# Unfolding ARF and ARL GTPases: from biophysics to systems-level insights

**DOI:** 10.3389/fmolb.2025.1712544

**Published:** 2025-12-09

**Authors:** Laura Quirion, Regina Strakhova, Matthew J. Smith, Jean-François Côté

**Affiliations:** 1 Montreal Clinical Research Institute (IRCM), Montréal, QC, Canada; 2 Molecular Biology Programs, Université de Montréal, Montréal, QC, Canada; 3 Institute for Research in Immunology and Cancer, Université de Montréal, Montréal, QC, Canada; 4 Department of Pathology and Cell Biology, Université de Montréal, Montréal, QC, Canada; 5 Department of Medicine, Université de Montréal, Montréal, QC, Canada; 6 Department of Anatomy and Cell Biology, McGill University, Montréal, QC, Canada

**Keywords:** ARF, ARL, GTPase, high-throughput, biophysics, BioID, ARL14, ARL10

## Abstract

Advanced technologies to study protein biophysics, mRNA expression and protein-protein interactions at high throughput in physiological or pathological contexts are reshaping our view of the ARF family of GTPases. Most current knowledge arises from work on the classical members ARF1 and ARF6, with many ARF-like proteins (ARLs) remaining poorly characterized. Recent findings suggest that several ARLs deviate from the binary molecular switch paradigm, instead exhibiting atypical biochemical properties, highly restricted tissue-specific expression patterns, specialized subcellular localizations, and unique interaction networks. These observations raise fundamental questions about the breadth of ARF family functions, mechanisms that regulate them, and their potential impact on cellular and organismal biology. In this review, we highlight emerging insights into atypical ARF members, outline unresolved questions, and discuss how expanding our understanding beyond the classical ARF members could shed light on their unique roles in health and disease.

## 1 Introduction

**FIGURE 1:**
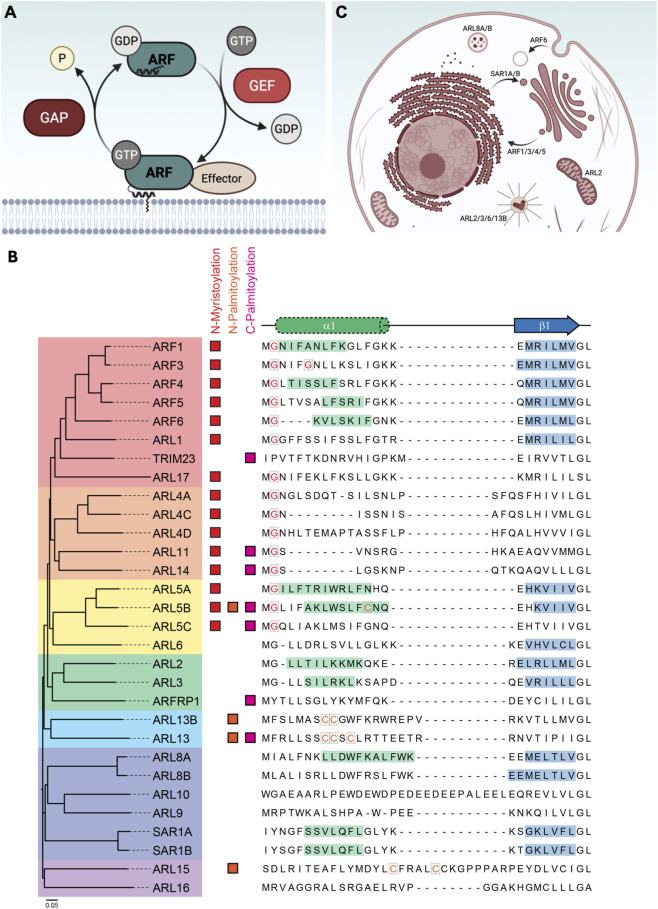
Phylogeny, N-terminal diversity and functions of the ARF family of GTPases. **(A)** Schematic of ARF cycling mediated by GEFs and GAPs. **(B)** Dendogram of the ARF/ARL family (left) establishes related subfamilies of GTPases (coloured boxes). The amino acid sequences of the N-terminal regions from these proteins are shown on the right. JPred secondary structure predictions reveal the presence or absence of an N-terminal helix (green) (Drozdetskiy et al., 2015). The β1 strand (blue) is a typical starting structural motif for RAS subfamily GTPases. Boxed residues represent potential post-translation modification sites, with lipidation characteristically occurring on Gly (myristoylation) or Cys (palmitoylation) residues. Potential sites of modification were determined using GPS-Lipid (Xie et al., 2016). **(C)** Overview of well-known ARFs and ARLs functions.

Small guanosine triphosphatases (GTPases) function as molecular switches that cycle between an inactive GDP-bound state and an active GTP-bound state. This cycling is regulated by guanine nucleotide exchange factors (GEFs), which catalyze GDP release to allow GTP binding, and by GTPase activating proteins (GAPs), which accelerate intrinsic GTP hydrolysis, converting GTP to GDP with the release of an inorganic phosphate (Bourne et al., 1991; Colicelli, 2004) (Figure 1A). The human ADP-ribosylation factor (ARF) family is a branch of the RAS superfamily of GTPases that comprises ∼30 members: 5 classical ARFs, 2 secretion-associated RAS-related proteins (Nachury et al., 2007), 22 ARF-like proteins (ARLs), and the tripartite motif-containing protein 23 (TRIM23) (Gillingham and Munro, 2007; Sztul et al., 2019). Phylogenetic analyses trace the origin of these proteins to the last common eukaryotic ancestor, which already encoded 15 ARF-related genes (Jackson et al., 2023). A distinctive feature of many ARF family proteins is an N-terminal extension that typically, though not always, folds into an amphipathic helix that is modified by myristoylation, palmitoylation, or acetylation (Figure 1B). This amphipathic helix is typically occluded in the GDP-bound state and exposed by GTP-mediated conformational changes, enabling membrane association. It is important to note that only 5 of the 22 ARF-like GTPases, namely ARL2, ARL3, ARL5A, ARL5B and ARL8A, were predicted to possess an N-terminal helix (Figure 1B). Furthermore, not all ARL proteins are predicted to contain residues that can undergo lipid modification (Figure 1B).

Functionally, classical ARFs are best known for their essential roles in membrane trafficking. ARF1 recruits different adaptors such as coat protein complex I (COPI), clathrin-associated adaptor protein complexes (AP-1, AP-3 and AP-4), and the Golgi-localized, γ-ear containing, ADP-ribosylation factor binding proteins (GGAs) to mediate vesicular transport between organelles (Sztul et al., 2019). Similarly, SAR1A and SAR1B regulate endoplasmic reticulum to Golgi vesicular trafficking through COPII complex recruitment (Van der Verren and Zanetti, 2023) (Figure 1C). ARFs are also involved in lipid composition remodeling by recruiting and activating a variety of lipid-modifying enzymes, and they are able to drive cytoskeletal changes through activation of RHO GTPases (Sztul et al., 2019).

By contrast, ARLs remain comparatively understudied despite evidence linking them to diverse processes such as lysosomal positioning (ARL8B) (Khatter et al., 2015), microtubule- and cilia-associated functions (ARL2, ARL3, ARL6, ARL13B) (Fisher et al., 2020) and mitochondrial fusion (ARL2) (Newman et al., 2017) (Figure 1C). This functional diversity, combined with distribution across multiple organelles and interplay between each other and other RAS superfamily members, underscores the complexity of the ARL family. Despite this, nucleotide cycling properties, effector engagement, and regulatory control of most ARLs remains poorly defined.

These foundations frame our current understanding of ARF GTPases. However, with the advent of new technologies and experimental approaches that interrogate proteins in their physiological environment, it is an exciting time to explore ARF function. Early findings are challenging long-standing paradigms and raise critical questions that will be the focus of this review.

## 2 Biophysics behind the ARF family of GTPases

As stated above, ARF and ARL proteins are thought to act as molecular “switches” by alternating between GDP-bound inactive and GTP-bound active states. The balance of conformational exchange is concentrated in two mostly unstructured regions, designated in all RAS proteins as switch I and switch II (Sztul et al., 2019). In the activated conformation, ARFs interact with downstream effectors that typically comprise an ARF-binding domain (ArfBD) or distinctive non-conserved domains able to recognize the GTP-loaded state, with switch I providing the principal contact surface (Chavrier and Menetrey, 2010).

**FIGURE 2:**
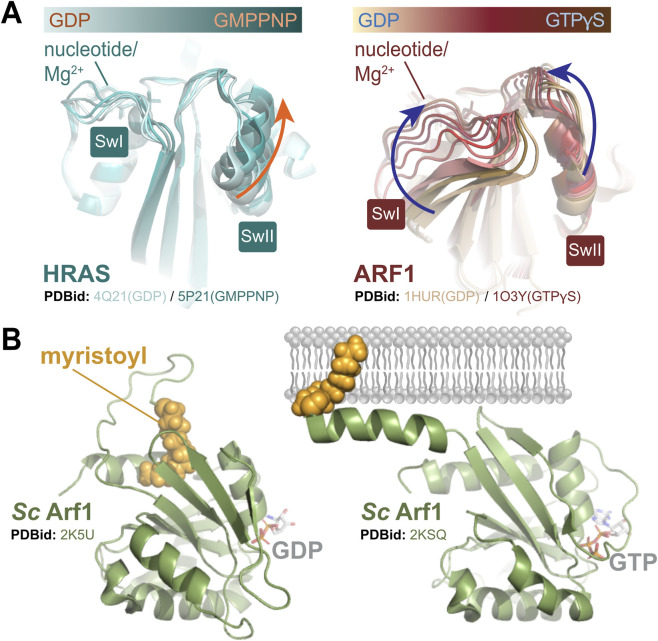
Biophysical properties of ARF GTPases. **(A)** Conformational exchange mediated by GTP binding in the classical HRAS GTPase (left) versus the human ARF1 GTPase (right). Ribbons diagrams are overlays of GDP- and GMPPMP/GTPγS-bound proteins with three (HRAS) or five (ARF1) simulated steps to represent motion. GTP-loading of HRAS results in a large shift in the position of the switch II region, while switch I maintains a similar arrangement. This does not assess changes in the dynamic movement of these regions. Structural rearrangements in ARF1 elicited by GTP-loading are more extensive, with both the switch I and II regions being significantly altered. In ARF GTPases, the switch I residues form a highly unique, additional β strand that runs antiparallel to β2. This is disrupted by GTP-binding, with switch I becoming unstructured and shifting over 14Å towards the γ-phosphate. The switch II region rotates approximately 25° towards the nucleotide binding pocket and forms a more extended helix. **(B)** The myristoylated N-terminal domain of ARF GTPases can impact nucleotide cycling in combination with lipid bilayers. As determined from NMR structures of full length, myristoylated yeast ARF1 (ribbons, green), when untethered to lipids and GDP-loaded the myristoyl group (yellow spheres representation) is buried in a hydrophobic groove between a C-terminal helix and the loop connecting β3 and β4, with the N-terminal domain of ARF1 remaining unstructured. Tethering of the GTPase to the membrane via the myristoyl group induces formation of a helix in the N-terminal domain that contributes to membrane binding. GTP-loaded ARF1 demonstrates significant reordering of the myristoyl binding pocket, suggesting it can no longer accommodate the lipid moiety.

There are few available atomic structures of ARF proteins, with only two members in the entire ARF/ARL families having structures in both the GDP- and GTP-bound conformations: ARF1 (GDP: PDB 7WQY, GTP: PDB 1HUR), and ARF6 (GDP: PDB 1E0S, GTP: PDB 2J5X). Compared with the characteristic structural transitions presented by the protooncogenic GTPases HRAS or KRAS, conformational exchange in ARF1 triggered by GTP loading is much more extensive and greatly impacts both the switch I and II regions (Figure 2A). As mentioned above, a distinctive structural and biochemical feature of ARFs that set them apart from other members of the RAS superfamily is an N-terminal amphipathic helix that is directly involved in controlling nucleotide cycling (Goldberg, 1998; Pasqualato et al., 2002). In the GDP-bound state, the N-terminal extension is mostly disordered and myristoylated on Gly2 with the lipid moiety occluded in a hydrophobic cavity between the interswitch region and a C-terminal helix (Antonny et al., 1997). Upon localization to the Golgi or plasma membrane, the lipidated N-terminal amphipathic helix is released and the myristoyl moiety inserted into the lipid bilayer (Figure 2B). This should permit GEF regulatory proteins to associate with ARFs and release GDP, which is rapidly exchanged for GTP due to its 10-fold increased abundance in cells (Sztul et al., 2019). The N-terminal helix, myristoyl group, membrane contact sites, and nucleotide binding pocket are believed to be tightly coupled in ARF GTPases, though much of our current understanding comes from work on ARF1 [(Amor et al., 1994; Seidel et al., 2004; Duijsings et al., 2009; Karandur et al., 2017)]. It is likely, however, that ARFs do not share identical biophysical and enzymatic properties. Structural studies have revealed the GDP-bound forms of ARF6 and ARF1 exhibit highly distinct conformations, particularly in the switch regions (Pasqualato et al., 2001). This may explain their functional divergence including differences in nucleotide exchange kinetics, lipid binding and GEF specificity (Jackson and Bouvet, 2014; Sztul et al., 2019). Intriguingly, structures of ARF1 and ARF6 in the activated state are highly similar (Pasqualato et al., 2001). Recent research has also revealed pressure-induced formation of a classical molten globule ensemble in ARF1-GDP, with increasing pressure favoring a switch transition (Koduru et al., 2024). This provides strong support for the concept of a molten globule playing a central mechanistic role in the nucleotide switch and may serve as an archetype for ARF biophysics.

While the ARF subclade appears to exhibit a “classical” small GTPase switch function, this is not true of the larger ARL subfamily. There are few data describing the biochemical activity of most members of the ARL clade, exemplified by no ARL proteins having both GDP- and GTP-bound structures solved to-date, but most data suggest the family exhibits a variety of aberrant biochemical properties (Bhamidipati et al., 2000; Gotthardt et al., 2015; Ivanova et al., 2017; Quirion et al., 2024; Zhang et al., 2016). Atypical or “pseudoGTPases” are now being identified throughout the RAS superfamily (Stiegler and Boggon, 2020). These are presumed small G-proteins that diverge in function from canonical RAS GTPases yet retain crucial cellular activities. While classical G-proteins act as molecular switches, cycling between GTP-bound active and GDP-bound inactive states, pseudoGTPases generally lack one or more motifs required for nucleotide binding and/or hydrolysis, or otherwise exhibit variations in amino acid composition that impair canonical activity (Stiegler and Boggon, 2020). Only recently have these proteins started to challenge the traditional paradigm of GTP-dependent effector binding and signaling. Multiple members of the RAS superfamily are now designated as pseudoGTPases, falling into distinct functional classes based on abnormal nucleotide interactions and/or cycling. These include: class I, which neither bind nucleotide nor hydrolyze GTP such as RHOBTB1/2 (Berthold et al., 2008; Chang et al., 2006); class II, which binds but cannot hydrolyze GTP including the RND proteins and RhoH (Guasch et al., 1998; Nobes et al., 1998); and class III, which retain both binding and hydrolysis activity but exhibit abnormal cycling or altered structural conformations, as observed with RGK GTPases, RHOT1/2 (MIRO1/2), and MRAS (Beguin et al., 2007; Bernal Astrain et al., 2025; Fransson et al., 2006). Structurally, known pseudoenzymes maintain the overall RAS-like fold, established by several crystal structures showing near-complete conservation of the core G-domain (RND3: PDB 4BG6, MIRO1: PDB 6D71 and 5KU1, MRAS: PDB 1 × 1R). Notable deviations are observed, however, in the switch I and II regions that classically mediate conformational exchange in response to nucleotide. Altered dynamics in these regions are undoubtedly coupled to defects in GDP/GTP binding or enzymatic activity.

Existing data suggest many ARL proteins have deviated from classical GTP/GDP cycling (Hillig et al., 2000; Burd et al., 2004; Bowzard et al., 2007). This includes ARL10, ARL11, ARL13, ARL14, ARL15 and ARL16, which all exhibit altered biochemical and/or structural properties. The use of multiple biophysical approaches has been instrumental in demonstrating that these GTPases are atypical, providing crucial knowledge to their unique biochemical mechanisms. First, amino acid alignments of ARL G-domains reveal several (ARL10, ARL13A/B, ARL15, and ARL16) lack an archetypally conserved glutamine in the switch II region, normally essential for GTP binding and hydrolysis (Colicelli, 2004). Insights from atomic structures determined by X-Ray crystallography pinpoint several further differences: less structured α-helix and an elongated loop were observed at the N-terminus of ARL3-GDP (PDB 1FZQ) (Hillig et al., 2000). ARL2 and ARL3 show altered binding to a cofactor Mg^2+^ ion, typically required for coordinating binding to nucleotides as well as GTP hydrolysis (Hillig et al., 2000; Zhang et al., 2009). This is due to the presence of a lysine residue at position 54, with the positively charged side chain disrupting binding to Mg^2+^ via electrostatic repulsion. Together these observations suggest that ARL2 and ARL3, additionally to GEFs, may require specific effector interactions to adopt a fully active conformation (Hillig et al., 2000; Zhang et al., 2016).

While structure determination can provide invaluable insight to ARL function, it provides information on only highly populated conformations and does not inform on the kinetics of nucleotide cycling (or lack thereof). Together with long-established approaches that monitor nucleotide binding, Nuclear Magnetic Resonance (NMR) spectroscopy has recently driven investigation of ARF/ARL conformational dynamics in solution, resolving previously undetermined properties of some ARLs that are inconsistent with standard switch-like behavior. Isotopically labelled ARL14 shows no detectable binding to GDP or GTP, revealing the protein is unable to undergo traditional nucleotide cycling (Quirion et al., 2024). Indeed, ARL14 appears partially unfolded in solution and fails to adopt a stable nucleotide-bound conformation *in vitro*. Its closest family member by homology is ARL11, yet this small GTPase can bind GTP with nanomolar affinity and adopts a more ordered structural conformation upon GTP loading (Quirion et al., 2024). Unlike classical RAS proteins which bind GTP and GDP with similar tight affinities, however, ARL11 binds GDP with only micromolar affinity and thus has a >1,000-fold preference for GTP (Quirion et al., 2024). This suggests the ARL11 protein is constitutively GTP-bound in cells and utilizes alternative means of regulation distinct from nucleotide cycling. A more distally related family member, ARL15, was also purified from bacteria in an apo state (no nucleotide bound, as with ARL14) but adopts a more ordered structure despite the absence of nucleotide (Mahbub et al., 2023).

In addition to NMR, small-angle X-ray scattering (SAXS) has enabled the analysis of overall molecular shape and conformational state of small G-proteins in solution, revealing that some pseudoGTPases maintain a stable, compact form that does not change upon addition of nucleotide. SAXS data established that apo-ARL15 is structured in solution yet does undergo major conformational change upon addition of either GDP or GTP, with transitions generally localized to the N-terminal extension (Saini et al., 2024). Further SAXS data on ARL GTPases should provide valuable insight to their dynamic association with nucleotides.

Finally, as it has been observed for ARF1 at the Golgi membrane or ARF6 at the plasma membrane, ARLs have a spatial aspect to their regulation (Peters et al., 1995). It has been shown that myristoylated ARL1 is regulated by PI(4,5)P2, whereby the lipid stabilizes the association of the protein with membrane bilayers (Seidel et al., 2004). Furthermore, the exchange of ARFRP1 and ARL14 N-terminal region led to a switch in their respective localization (Yang et al., 2020). More research is required to determine how lipids impact ARL nucleotide binding and/or enzymatic activity, and multiple experimental approaches (e.g. NMR and cryo-EM) are now able to explore these questions. It is clear that biochemical activity of many atypical ARF-like GTPases has diverged significantly from archetypal RAS GTPases, and many should be considered pseudoGTPases. This challenges traditional paradigms of GTPase function and highlights the need to investigate each family member individually, rather than assuming a conserved mechanism.

## 3 High througput approaches to study ARF and ARL GTPases

**FIGURE 3:**
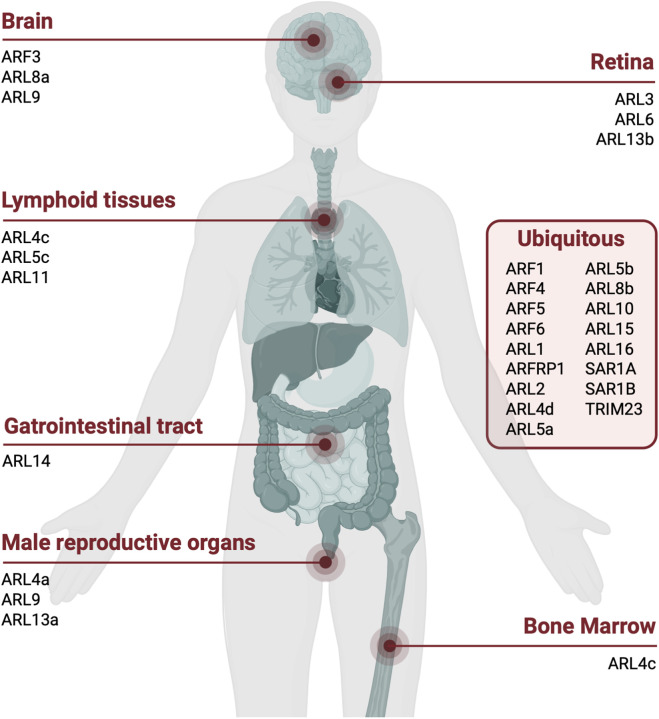
ARF and ARL GTPases expression in human tissues. Representation of ARFs and ARL distribution across human tissues based on mRNA expression levels extracted from the Human Protein Atlas project. The figure was created in BioRender.

Recent technical advances have greatly expanded our ability to study cellular processes at both higher throughput and increased resolution. At the transcriptomic level, single-cell RNA-sequencing (scRNA-seq) datasets are now widely available, offering a broad overview of gene expression patterns across organs and cell types. Mapping the mRNA expression of genes coding for understudied ARFs can provide important clues to their physiological function, particularly when expression is restricted to specific cell types. Although most ARF family members show low tissue specificity, scRNA-seq deposited into the Human Protein Atlas (proteinatlas.org) highlight distinct expression patterns for several ARFs and ARLs, notably in the brain (ARF3, ARL8a, ARL9), retina (ARL3, ARL6, ARL13b), male reproductive organs (ARL4a, ARL9, ARL13a), bone marrow (ARL4c), lymphoid tissues (ARL4c, ARL5c, ARL11), and the gastrointestinal tract (ARL14) (Figure 3) (Karlsson et al., 2021).

ScRNA-seq data suggest that *ARL14* is expressed in gastric mucus-secreting cells, distal enterocytes, Paneth cells, proximal enterocytes, intestinal goblet cells and enteroendocrine cells (Karlsson et al., 2021). In a murine model, CRISPR-mediated endogenous tagging of Arl14 and subsequent Western blot analysis of collected organs confirmed its restricted gastrointestinal expression, with specific expression in the stomach and intestine tissues (Quirion et al., 2024). ARL10, another poorly characterized member of the ARF family, shows enhanced expression specifically in oligodendrocyte precursor cells, oligodendrocytes, inhibitory neurons, and excitatory neurons (Karlsson et al., 2021). This type of single cell resolution transcriptomic data provides valuable clues into the likely functions of atypical ARFs/ARLs in their native environment, information that is often absent in more simple overexpression systems.

At the proteome level, proximity labelling approaches have recently been applied to systematically define the ARF family interactome (Li et al., 2022; Quirion et al., 2024). These methods identify proteins in the immediate vicinity of a bait of interest, capturing not only stable but also weak, transient, and/or context-dependent interactions. The two most widely used systems are based on engineered enzymes, ascorbate peroxidase (APEX) or the *E. coli* derived biotin ligase BirA, each of which can be adjusted to label over assorted time scales ranging from 1 min to 24 h (Dionne and Gingras, 2022). Short labelling windows provide snapshots of interaction under specific conditions (e.g. growth factor stimulation, adhesion, drug treatment), whereas longer labelling times yield more comprehensive maps that include rare or transient interactions in addition to proteins that are resident of specific subcellular regions.

The ARF family has now been studied using both long-exposure BioID (Quirion et al., 2024) and rapid labeling with miniTurbo (∼15 min) (Li et al., 2022), with these approaches collectively providing a comprehensive and dynamic view of the ARFome. In one study, BioID followed by mass spectrometry was performed on 28 ARF family members (excluding TRIM23) in HeLa and HEK293 cells, identifying ∼4600 high-confidence proximal interactions (Quirion et al., 2024). In parallel, a miniTurbo approach applied to 25 ARF family members (excluding ARL4A, ARL5C, ARL9 and ARL13A) in HEK293 cells detected ∼1600 proximity interactors. Collectively, they elucidate interactomes for nearly the entire ARF/ARL family. Future efforts are required to stratify these interactors into regulatory GEFs and GAPs versus effector proteins, and to validate all potential candidates biochemically. Nonetheless, these resources already refine our understanding of the best characterized ARFs and open new avenues for probing atypical ARLs. Importantly, BioID data has also yielded insights into subcellular localization, revealing multiple pools of ARFs/ARLs at distinct cellular compartments. Determining the functional purpose of these spatially separated pools remains a key challenge in the future. Overall, the emergence of high-throughput transcriptomic and proteomic methods is reshaping our understanding of the ARF GTPase family, where only a handful of members had been thoroughly studied until recently.

## 4 Roles and localization of two atypical ARLs

As with biophysical studies, current knowledge of ARF GTPase biology derives largely from work on classical ARFs, SARs, and a handful of ARLs. Consequently, most ARL proteins remain poorly characterized, representing a major gap in our understanding of this family. This section focuses on two atypical ARLs whose localization, function, and disease associations are still poorly explored.

ARL14 is one of the most understudied members of the ARF family. Analyses of “The Cancer Genome Atlas” (TCGA) lung cancer cohorts revealed that ARL14 expression is upregulated in non-small cell lung cancer (NSCLC), including lung squamous cell carcinoma (Armbruster and Luschnig, 2012) and lung adenocarcinoma (LUAD) (Zhang et al., 2021; Ma et al., 2025; Ding et al., 2022; Guo et al., 2019) with elevated ARL14 expression correlated with poor patient prognosis. These observations have been corroborated by immunohistochemistry in patient samples and the NSCLC cell lines A-549, PC9 and NCI-H1299. Functional studies also support a role for ARL14 in tumorigenesis. Genetic depletion of ARL14 using siRNA was shown to significantly reduce cell viability and proliferation, as well as migration and invasion (Ma et al., 2025; Guo et al., 2019). These data position ARL14 as an attractive therapeutic target in NSCLC. Beyond lung cancer, genetic variation at the *ARL14* locus has been linked to susceptibility or resistance to malaria, as demonstrated by the presence of single nucleotide polymorphisms in the *ARL14* gene (Adjemout et al., 2024).

Though the impact of ARL14 in human disease is beginning to be resolved, only a handful of studies have addressed its molecular function. A genome-wide, flow cytometry-based RNAi screen first identified ARL14 as a candidate regulator of MHC-II transport to the plasma membrane (Paul et al., 2011). Subcellular localization studies further revealed ARL14 at the plasma membrane and in vesicles, confirmed by GFP-tagged ARL14 expressed in HeLa cells (Yang et al., 2020). Some of these vesicles were positive for EEA1, suggesting partial enrichment of ARL14 in early endosomes. Mechanistically, the N-terminal region of ARL14 (aa 2–17) was essential and sufficient for membrane association and appropriate localization (Yang et al., 2020). A glycine at position 2 of ARL14 undergoes myristoylation or palmitoylation (Figure 1C), and its mutation abrogates ARL14 membrane targeting (Yang et al., 2020). With respect to upstream regulation, PSD4 was identified as a potential GEF, based on RNAi screening of SEC7 domain-containing proteins that enhance MHC-II transport, and an *in vitro* GTP loading assay supported this biochemical activity (Paul et al., 2011). No additional regulatory proteins have been reported to date. As for ARL14 effectors, ARF7EP was identified as a direct interactor using a yeast-two-hybrid approach, and this was validated by co-immunoprecipitation and co-localization (Paul et al., 2011). However, there is currently no data indicating these proteins are expressed in the same cell types. BioID studies identified phospholipase D1 (PLD1; an enzyme that hydrolyze phosphatidylcholine into phosphatidic acid, a signaling lipid, and choline) as an ARL14 effector. Insights from ARL11, ARL14’s closest homolog that also interacts with PLD1, suggest that this interaction is mediated through the loop region of PLD1 between two conserved HxKxxxxD (HKD) motifs (Li et al., 2022). Functionally, ARL14 activates PLD1 both *in vitro* (activity assays with purified proteins) (Bowling et al., 2020) and *in cellulo* (using the IMPACT assay (Imaging PLD Activity with Clickable Alcohols via Transphosphatidylation)) (Bumpus et al., 2020; Quirion et al., 2024). Finally, proximity labeling also revealed that ARL14 associates with the endosomal SNX-BAR sorting complex for promoting exit-1 (ESCPE-1), composed of dimers of SNX1/2 and SNX5/6. Supporting this, loss of ARL14 impaired recycling of the cation-independent mannose-6-phosphate receptor (CI-MPR), a known ESCPE-1 cargo, impairing trafficking from endosomes to the trans-Golgi network (Quirion et al., 2024). Together, these findings outline a growing but still fragmentary picture of ARL14 biology, suggesting it plays important roles in membrane trafficking through regulation of endosomal transport and PLD1 signaling.

ARL10 was recently characterized as a distinctive member of the ARF family, notable for its unusually long N-terminal extension that harbors a transmembrane domain essential for mitochondrial targeting (Figure 1C). BioID-based proximity labeling, integrated with the Human Cell Map, predicted this mitochondrial localization as well as peroxisomal targeting, which was subsequently confirmed by co-immunostaining (Quirion et al., 2024). A mitochondrial localization is supported by inclusion of ARL10 in the human mitochondrial high-confidence proteome (MitoCoP) (Morgenstern et al., 2021), and this GTPase was also detected in the proteome of TOMM20-positive mitochondrial-derived vesicles (Konig et al., 2021). Although a specific role for ARL10 remains to be clarified, gene ontology analyses point to potential roles in mitophagy, protein localization to peroxisomes, and TORC2 signaling (Quirion et al., 2024). To-date, the direct effectors and regulatory mechanisms of ARL10 remain unknown, highlighting the early stage of our understanding of this atypical ARF family member.

## 5 Importance of better understanding atypical GTPases

A wide array of experimental approaches consistently uncovers atypical features of ARL GTPases that deviate from the classical molecular switch paradigm, underscoring complexity in both their biology and molecular function, suggesting a need to reconsider how this GTPase family might function.

### 5.1 Functional variations: nucleotide binding, sequence and structural considerations

Biophysical studies of ARLs have taught us the importance of validating nucleotide binding, rather than relying solely on RAS-inspired point mutations to drive constitutive GDP- or GTP-bound states. Such substitutions do not consistently produce the expected biochemical outcome and can yield misleading results. For example, although ARL14 retains the conserved switch II glutamine required for GTP hydrolysis, a Q68L mutant, analogous to the KRAS Q61L activating mutation, shows the identical nucleotide binding profile as WT ARL14 (Quirion et al., 2024). Consequently, the localization and interactors reported for this mutant may not reflect genuine active-state signaling. NMR and ITC studies further revealed that ARL14 has unusually low affinity for both GDP and GTP, raising the possibility that binding is stabilized *in cellulo* through membrane association or interactions with regulators or effectors (Quirion et al., 2024). The designation of PSD4 as a candidate GEF for ARL14, based on *in vitro* α^32^P-GTP loading assay, will require validation in more physiologically relevant systems. Structural insights will be crucial to determine whether ARL14 undergoes conformational changes upon binding of GTP, partner proteins and/or lipids. Another possibility is that it may function as a pseudoGTPase. How pseudoGTPase signaling might be regulated remains an open question, but could involve transcriptional control, post-translational modification, or competitive binding interactions outside the classic nucleotide binding pocket.

Another structural aspect to consider is the purpose of the N-terminal α-helix. Classical ARFs absolutely require the N-terminal α-helix for proper nucleotide loading while some ARLs seem to bind nucleotides independently of the region. Even though the helical structure is not always maintained, the conservation of this N-terminal region in ARLs indicate its biological function beyond nucleotide loading. It has been shown that this extension can play a role in protein and cargo trafficking for ARL3, ARL8A, ARL8B, ARL13B and possibly other GTPases (Hanke-Gogokhia et al., 2016; Hofmann and Munro, 2006; Palicharla et al., 2023). Roughly half of ARLs retain the conserved glycine residue that undergoes myristoylation in ARFs, even though some of them lack a predicted N-terminal helical structure (Figure 1C). The other half generally lack glycine or cystein residues hat could serve as potential sites for lipid modification. Thus, ARL proteins that comprise very low nucleotide affinity may rely on nucleotide stabilization by binding partners or membrane association, that may require the N-terminal extension and/or lipidated residues, or on unknown regulatory mechanisms besides classical GTP/GDP cycling. Defining these interactions and mechanisms will be key to determining whether they act as *bona fide* GTPases or pseudoGTPases.

### 5.2 Non-canonical effector binding

Unlike RHO and RAB GTPases, which are sequestered in their GDP-bound state by GDP-dissociation inhibitors (GDIs) in the cytosol, ARFs possess an intrinsic “self-inhibition” mechanism provided by the N-terminal amphipathic helix, preventing membrane association and effector engagement. This paradigm is exemplified by ARF1, yet the mechanism is not universal. In the case of ARF6, data suggest it can interact with proteins in its GDP-bound form, for example with the ubiquitin carboxyl-terminal hydrolase 6 (USP6) (also known as TRE17 or TRE2). This interaction facilitates the recruitment of ARF6-GDP to the plasma membrane, where it can subsequently be activated by GEFs (Martinu et al., 2004). For atypical ARLs, including ARL10 that possess a transmembrane domain, self-inhibition is unlikely to apply, raising the possibility that GDP-bound conformations may also mediate effector interactions. Such deviations again challenge the binary switch model and expand the potential signaling mechanisms of ARFs/ARLs.

### 5.3 GTPase crosstalk

Previous studies clearly highlight a complex interplay within the ARF family but also with other GTPases. For example, ARL1 influences the recruitment and activation of ARF1 and ARF3 in RAB4A positive endosomes by the recruitment of the ARF GEFs BIG1 and BIG2 (D'Souza et al., 2014). Another example is a role for ARF1 in regulating RAC1 signaling: ARF1 is required for membrane targeting of RAC1 and its effector IRSP53, an adaptor protein linking RAC1 to the WAVE regulatory complex, crucial for lamellipodia formation and cell migration (Lewis-Saravalli et al., 2013). With the recent and future definition of ARF, RHO, RAB and RAS GTPase interactomes, the integration of these datasets offers a unique opportunity to identify intersection points within the broader “GTPasome,” highlighting shared effectors, regulatory nodes, and pathways. The consolidation of these networks into a global signaling hub should provide a comprehensive view of small GTPase biology and reveal unexpected interplay across families.

### 5.4 Context dependence of ARF/ARL interactions

High-throughput approaches have revealed both tissues-enriched expression of specific ARF/ARLs and the complexity of their interactomes. However, most interaction data have been generated in heterologous systems such as HEK293, raising questions about their relevance to specialized cell types with distinct proteomes. However, the integration of this data can provide clues into the biological roles of ARF and ARL GTPases. For example, combining ARL14’s restricted expression in specialized gastro-intestinal cells with its known role in endosomal transport discovered using proteomic approaches suggest it may contribute to the secretory pathways of these cells. While there is no doubt that discovery proteomics approaches are powerful, subsequent work to resolve ARF signaling networks should include study of these tissue-enriched proteins in a more native context. Endogenous CRISPR-tagging offers an appealing solution, enabling protein study under the control of native promotors and at physiological expression levels. Such approaches will be key to resolving context-specific signaling mechanisms.

### 5.5 Pathophysiological relevance

Ciliopathies are a prominent group of human disease. Autosomal recessive mutations in *ARL3* or *ARL13B* cause Joubert Syndrome (Glass et al., 1993), while autosomal recessive mutations in *ARL6* (also known as BBS3) lead to Bardet–Biedl Syndrome (Forsyth and Gunay-Aygun, 1993). Neurodevelopmental disorders such as periventricular nodular heterotopia have been linked to ARF1 (de Sainte Agathe et al., 2023). Beyond genetic diseases, aberrant expression of ARFs/ARLs is increasingly recognized in cancer, where they promote tumor progression. ARL14, normally expressed in the gastrointestinal tract, is ectopically upregulated in NSCLC and correlated with poor prognosis, raising questions about transcriptional regulation and selective advantages in lung cancer cells. Such cases highlight the therapeutic potential of targeting ARF/ARL GTPases, their regulators, or effectors.

In conclusion, small GTPases functions within highly dynamic signalling networks, where even minor perturbations in expression, cycling, or protein-protein interactions can dramatically alter cellular outcomes. To fully appreciate the biology of atypical ARLs, we must move beyond *in vitro* assays and overexpression systems and interrogate their behaviour *in cellulo* and *in vivo* under native conditions. Comprehensive studies that integrate structural, biochemical, and cell biological approaches will be critical for defining whether these proteins function as *bona fide* molecular switches, and in some cases, as pseudoGTPases. Such insights will not only clarify their fundamental roles but may also open new avenues for therapeutic intervention in both genetic disorders and cancers.
